# Soluble MOG35-55/I-A^b^ Dimers Ameliorate Experimental Autoimmune Encephalomyelitis by Reducing Encephalitogenic T Cells

**DOI:** 10.1371/journal.pone.0047435

**Published:** 2012-10-15

**Authors:** Yeli Gong, Zhigang Wang, Zhihui Liang, Hongxia Duan, Lichen Ouyang, Qian Yu, Zhe Xu, Guanxin Shen, Xiufang Weng, Xiongwen Wu

**Affiliations:** Department of Immunology, Tongji Medical College, Huazhong University of Science and Technology, Wuhan, China; Escola Paulista de Medicina - UNIFESP, Brazil

## Abstract

The MOG35-55 peptide-induced experimental autoimmune encephalomyelitis (EAE) model in C57BL/6 mice is a useful animal model to explore therapeutic approaches to T cell-mediated autoimmune diseases because the dominant T-cell epitope(s) have been defined. It is rational that antigen-specific immunosuppression can be induced by using MHC-peptide complexes as specific TCR ligand(s) that interact with autoreactive T cells in the absence of co-stimulation. In this study, a soluble divalent MOG35-55/I-A^b^ fusion protein (MOG35-55/I-A^b^ dimer) was constructed to specifically target the autoreactive CD4^+^ T cells in the EAE mouse. Intraperitoneal administration of the MOG35-55/I-A^b^ dimer significantly delayed and ameliorated EAE symptoms by reducing EAE-related inflammation in the mouse CNS and reducing encephalitogenic Th1 and Th17 cells in the peripheral lymphoid organs. We observed that dimer intervention at a concentration of 1.2 nM suppressed MOG35-55 peptide-specific 2D2 transgenic T cells (2D2 T cells) proliferation by over 90% after *in vitro* activation with MOG35-55 peptide. The mechanisms involved in this antigen-specific dimer-mediated suppression were found to be downregulated TCR-CD3 expression as well as upregulated expression of membrane-bound TGF-β (mTGF-β) and IL-10 suppressive cytokines by the autoreactive CD4^+^ T cells. Collectively, our data demonstrates that soluble divalent MHC class II molecules can abrogate pathogenic T cells in EAE. Furthermore, our data suggests that this strategy may provide an efficient and clinically useful option to treat autoimmune diseases.

## Introduction

Multiple sclerosis (MS) and its animal model, experimental autoimmune encephalomyelitis (EAE), are autoimmune diseases mediated by myelin-reactive CD4^+^ T cells targeting myelin-producing cells of the CNS [1]. Autoreactive CD4^+^ T helper (Th) cells, especially IFN-γ-producing Th1 and IL-17-producing Th17 cells are key players in encephalitogenic pathology [2], [3]. It is speculated that classical Th1 cells participate in the initial inﬂammatory phase of EAE and that IL-17-producing Th17 cells are generated rapidly in the CNS and may be critical to target organ damage [4]. EAE can be induced in genetically susceptible mouse strains by immunization with myelin antigens, including myelin basis protein (MBP), proteolipid protein (PLP) and myelin oligodendrocyte glycoprotein (MOG). MOG35-55 peptide is the major immunodominant epitope of MOG; it has been identified as an agonist of encephalitogenic T cells in the C57BL/6 mouse model [5]. MOG35-55 peptide-induced EAE in the C57BL/6 mouse serves as an animal model to explore therapeutic approaches to the MS, and the transgenic 2D2 mouse of a C57BL/6 background expressing Vα3.2/Vβ11 TCR with specificity for MOG35-55 peptide facilitates this exploration [6].

Antigen-specific immunomodulation is a critical goal for immune intervention to be able to inhibit the pathogenic inflammatory reactions that underlie many autoimmune diseases. It is well established that co-stimulatory accessory molecules, such as B7/CD28, must be triggered simultaneously with the TCR-MHC interaction for sufficient T-cell activation. Triggering the TCR-MHC interaction in the absence of co-stimulatory signals leads to tolerant and unresponsive antigen-dependent T cells, referred to as clonal anergy [7]; therefore, a soluble MHC molecule that binds only to the specific TCR has the potential to induce this anergic tolerance. Accordingly, various forms of recombinant MHC molecules that serve as a new generation of immunospecific T cell modulators have recently been developed. These modulators have therapeutic potential in immune-mediated pathologies, such as organ allograft transplants and autoimmune diseases [8], [9], [10], [11], [12]. One of the most successful recombinant MHC molecules is the recombinant T-cell receptor ligand (RTL), which is a single polypeptide chain consisting of the α1 and β1 domains of MHC class II molecules genetically linked to autoantigenic peptides. RTLs have been shown to signal directly through TCR as partial agonists, preventing and treating EAE in different animal models. In particular, RTL1000 has been shown to reverse clinical paralysis in mice developing EAE and is recently evaluated in a Phase 1 safety study [13], [14]. The success of this treatment suggests that recombinant MHC molecules may have therapeutic effects against autoimmune diseases.

As an alternative strategy for engineering soluble recombinant MHC complexes, dimeric MHC-Ig fusion proteins (MHC dimers) can be constructed by fusing the MHC chain with an IgG heavy chain or Fc fragment [15], [16], [17]. Specific peptides can be pulsed into the MHC groove to govern the TCR-ligand specificity for the targeted T cells. While low concentrations of MHC dimers have been demonstrated to mediate immunosuppression in autoreactive or alloreactive T cells *in vitro*, their immunotherapeutic potential has yet to be demonstrated further *in vivo* [8], [12], [17].

In this study, a soluble divalent I-A^b^/IgG fusion protein molecule (I-A^b^ dimer) pulsed with MOG35-55 peptide (MOG35-55/I-A^b^ dimer) was used to target the MOG35-55 peptide-specific T cells. The findings showed that autoreactive T cells in the MOG35-55 peptide-induced EAE model in C57BL/6 mice were specifically suppressed by this dimer *in vivo*; 4 doses (1 µg/dose) of the dimer caused delayed onset and significant amelioration of EAE symptom scores, accompanied by a remarkable decrease in the number of encephalitogenic Th1 and Th17 cells in the peripheral lymphoid organs. *In vitro* study showed the dimer also inhibited the 2D2 transgenic T cell proliferation and both Th1 and Th17 cells expansion in response to MOG35-55 peptide stimulation. The downregulation of CD3-TCR expression and the upregulation of suppressive cytokines production (IL-10 and mTGF-β) in the 2D2 T cells were possible mechanisms behind how the dimer could induce suppression of EAE autoreactive cells and amelioration of EAE symptoms. Overall, our study demonstrates that the soluble divalent MHC molecules have antigen-specific suppressive effects on autoreactive T cells, and suggests that this strategy may have applications in immunotherapy development for T cell-mediated autoimmune diseases.

## Results

### MOG35-55/I-A^b^ Dimer Inhibits the *in vitro* Proliferation of MOG35-55-specific T cells and Expansion of Th1 and Th17 Cells at Nanomolar Concentrations

In order to confirm that the soluble MOG35-55/I-A^b^ dimer was correct, ELISA and western blotting using specific antibodies were performed. The proposed conformation of the fusion protein is shown in Fig. S1B. The dimer secreted by the recombinant baculovirus-infected sf9 cells, containing the I-A^b^/IgG gene, was able to react with MHC class II (I-A/I-E)-specific antibody 2G9 or M5/114.15.2, and an IgG2b-Fc-specific antibody (Fig. S1C). This suggested that the I-A^b^ structure of the dimer was intact, as the 2G9 and M5/114.15.2 are both conformation-dependent antibodies. The protein A column-purified dimer demonstrated two bands with molecular mass corresponding to I-A^b^α-Fos-Fc (53 kD) and I-A^b^β-Jun (31 kD) in SDS-PAGE analysis under reducing conditions, and these bands were reactive with polyclonal antibodies specific for the Fos and Jun dimerization domains as revealed by western blotting (Fig. S1D). In addition, I-A^b^ dimers loaded with I-A^b^-restricted peptides, biotinylated-MOG35-55 as well as biotinylated-Mulv env145–158, were reactive to HRP-labeled streptavidin, suggesting these peptides can be effectively loaded onto I-A^b^ dimers (Fig. S1E).

**Figure 1:**
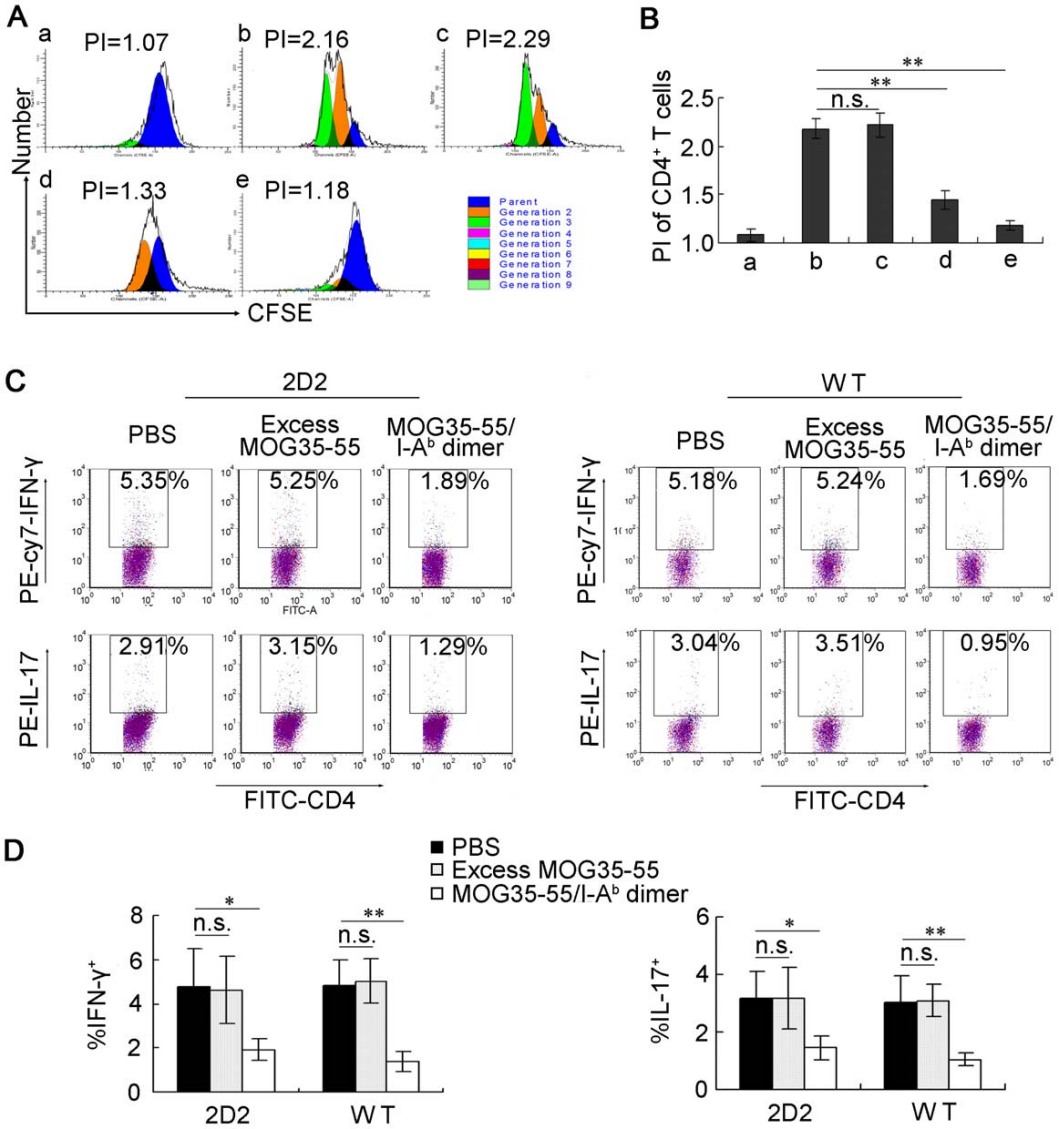
MOG35-55/I-A^b^ dimer inhibits the proliferation and pathogenic cytokine production of MOG35-55-specific T cells. (A–B) Splenocytes from 2D2 transgenic mice were labeled with CFSE and stimulated by MOG35-55 peptide in the presence of PBS, excess MOG35-55 peptide or MOG35-55/I-A^b^ dimer. After 3 days of co-culture, the cells were harvested and CD3^+^CD4^+^ T cells were gated for CFSE dilution assay. The proliferated cells are detected as the sub-population with low CFSE intensity compared to the parent cells, which do not proliferate. Various peaks with differing colors represent generations of proliferated cells. Group as follows: (a) Unstimulated. (b) Stimulated with MOG35-55 (25 µg/mL). (c) Stimulated with MOG35-55 (25 µg/mL) and excess MOG35-55 peptide in a concentration used to be loaded on the dimer. (d) Stimulated with MOG35-55 (25 µg/mL) in the presence of MOG35-55/I-A^b^ dimer (0.12 nM). (e) Stimulated with MOG35-55 (25 µg/mL) in the presence of MOG35-55/I-A^b^ dimer (1.2 nM). (A) Proliferation assay of 2D2 T cells from one representative experiment. (B) The proliferation index (PI) is presented as the mean ± SD from four separate experiments (n = 4). (C–D) The splenocytes from 2D2 or MOG35-55-immunized wild-type C57BL/6 mice (WT) were stimulated with MOG35-55 (25 µg/mL) for 5 days in the presence of PBS, excess MOG35-55 peptide or MOG35-55/I-A^b^ dimer, and IFN-γ and IL-17 expression were detected by Intracellular staining. (C) Representative dot plots for one 2D2 mouse and one WT mouse. (D) The mean values of percentage of IFN-γ or IL-17-producing cells are represented as the mean ± SD showing the differences (n = 4). *, *P<*0.05; **, *P<*0.01 and n.s., nonsignificant.

The effect of the soluble MOG35-55/I-A^b^ dimer on autoreactive T cells was first examined in a T-cell proliferation assay using splenocytes taken from the C57BL/6-background 2D2 transgenic mice. These 2D2 T cells have TCR specificity for the encephalitogenic MOG35-55 peptide, and could specifically respond to the MOG35-55 peptide stimulation *in vitro* [6]. After stimulation with MOG35-55 peptide for 3 days *in vitro,* the proliferation of 2D2 CD4^+^ T cells was analyzed by CFSE dilution assay. Proliferation magnitude was determined by proliferation index (PI). As expected, 2D2 CD4^+^ T cells exhibited vigorous proliferation with the stimulation of MOG35-55 peptide *in vitro*; the average PI of the CD4^+^ T cell reached 2.18±0.11 from four separate experiments. MOG35-55/I-A^b^ dimer, but not the excess MOG35-55 peptide, inhibited this autoreactive T-cell proliferation (Fig. 1A). Specifically, the presence of MOG35-55/I-A^b^ dimer significantly decreased the PI to 1.44±0.10 at 0.12 nM (*P<*0.01) and 1.18±0.05 at 1.2 nM (*P<*0.01) (Fig. 1B). Indeed, more than 90% inhibition of CD4^+^ T cells proliferation was achieved with MOG35-55/I-A^b^ dimer at the 1.2 nM concentration.

To further understand the effects of MOG35-55/I-A^b^ dimer on the function of 2D2 CD4^+^ T cells, intracellular staining for IFN-γ and IL-17 were performed after 5 days stimulation with MOG35-55 peptide. The results showed that MOG35-55/I-A^b^ dimer reduced the MOG35-55 peptide-induced expansion of IFN-γ-producing Th1 and IL-17-producing Th17 cells. After *in vitro* MOG35-55 peptide stimulation, the average percentage of IFN-γ-producing Th1 2D2 CD4^+^ T cells was 4.55%±1.44%; this decreased to an average of 1.91%±0.49% under intervention of MOG35-55/I-A^b^ dimer (*P*<0.05, n = 4) (Fig. 1C-D). IL-17-producing Th17 expansion *in vitro* was also reduced in response to MOG35-55 peptide stimulation under intervention of the dimer (1.44%±0.41% for the MOG35-55/I-A^b^ dimer intervention group vs. 3.17%±1.07% for the PBS control group, *P*<0.05, n = 4) (Fig. 1C-D). Similar results were also got from autoreactive T cells from wild-type (WT) C57BL/6 mice. Splenocytes from MOG35-55-immunized WT C57BL/6 mice were stimulated with MOG35-55 peptide. In the presence of MOG35-55/I-A^b^ dimer, percentages of Th1 and Th17 cells were significantly lower compared with PBS controls (dimer intervention group vs. PBS control: Th1%: 1.38%±0.49% vs. 4.94%±1.17% [*P<*0.01, n = 4]; Th17%: 1.05%±0.22% vs. 3.00%±0.47% [*P<*0.01, n = 4]) (Fig. 1C-D). The excess MOG35-55 intervention did not show any suppressive effect either to 2D2 T cells or WT cells. Taken together, these data indicate that MOG35-55/I-A^b^ dimer intervention can inhibit the expansion of autoreactive Th1 and Th17 cells *in vitro*.

### MOG35-55/I-A^b^ Dimer, but not the Excess MOG35-55 Peptide or Irrelevant I-A^b^ Dimer, Ameliorates Clinical Signs of EAE and Reduces CNS Inflammation

**Figure 2:**
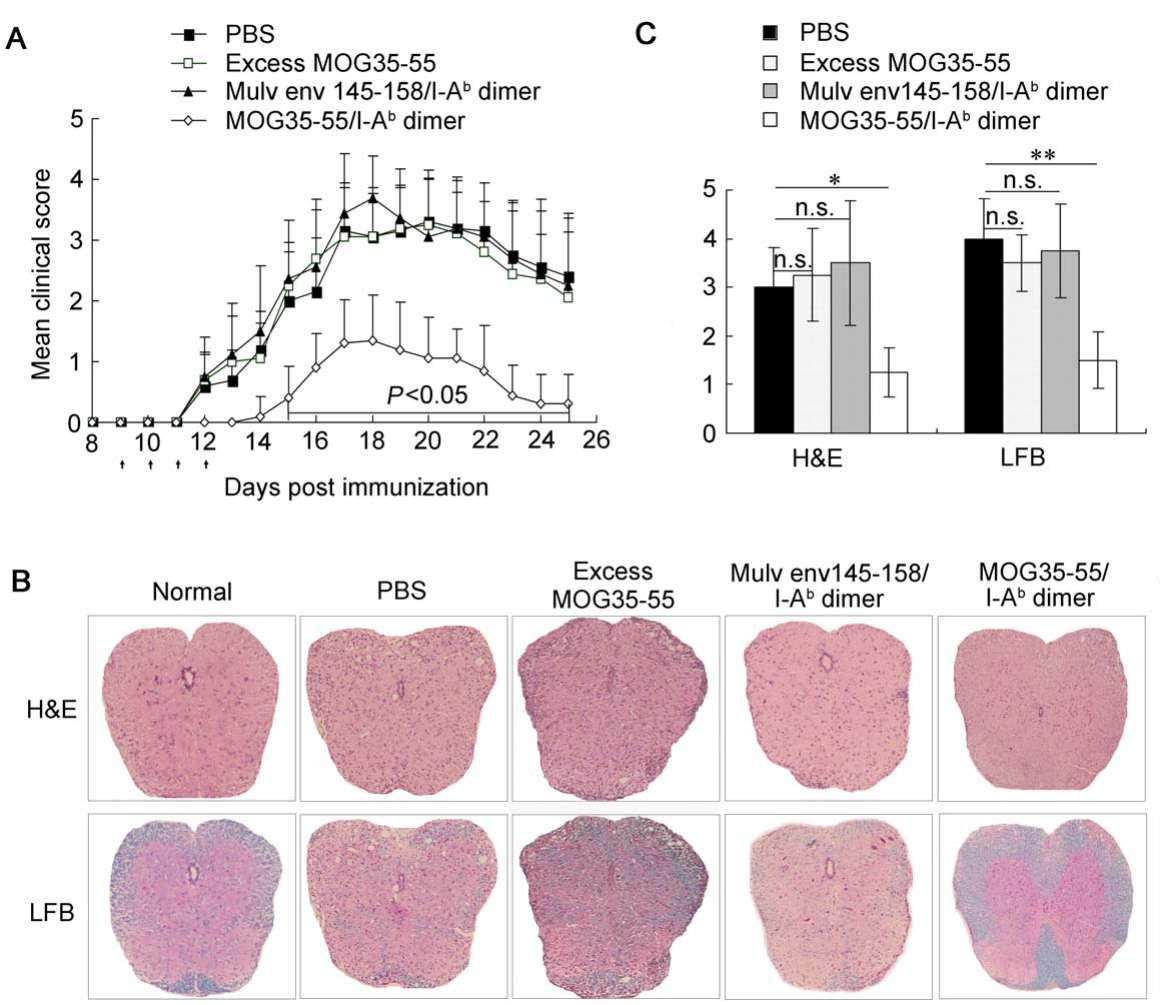
MOG35-55/I-A^b^ dimer delays onset and reduces severity of EAE *in vivo*. Female C57BL/6 wild-type mice were immunized with MOG35-55 to induce paralyzed EAE. PBS, excess MOG35-55 peptide, Mulv env145-158/I-A^b^ dimer (1 µg per mouse) or MOG35-55/I-A^b^ dimer (1 µg per mouse) was administered i.p. starting at day 9 post-immunization. Treatments lasted 4 days. (A) Data are expressed as mean clinical scores ± SD (n = 10 for PBS and MOG35-55/I-A^b^ dimer-treated groups, n = 8 for excess MOG35-55 and Mulv env145-158/I-A^b^ dimer-treated groups). Black arrows indicate i.p. injection. The mean clinical score in MOG35-55/I-A^b^ dimer-treated group is lowest (*P<*0.05), whereas there is no difference among PBS, excess MOG35-55 and Mulv env145-158/I-A^b^ dimer-treated groups. (B–C) Spinal cord sections obtained from the above four groups at day 25 post-immunization were analyzed for degree of inflammation by H&E and for demyelination by LFB (original magnification 200×). (B) The MOG35-55/I-A^b^ dimer-treated group showed minimal inflammatory cell infiltration and demyelination. One representative sample from each group is depicted. (C) Semi-quantitative analyses of inflammation and demyelination in spinal cords from four groups were conducted. Histopathological scores were determined as described in *Materials and Methods* and are presented as mean ± SD from 4 mice. Data presented are representative of two independent experiments. Differences between groups were assessed by Mann-Whitney U test. *, *P<*0.05; **, *P<*0.01 and n.s., nonsignificant.

To test the effects of soluble MOG35-55/I-A^b^ dimer *in vivo*, female C57BL/6 mice were first immunized with MOG35-55 peptide emulsified in adjuvant to induce active EAE. Upon onset of clinical signs of actively induced EAE (day 9 after immunization), mice were treated daily with the experimental dimer (MOG35-55/I-A^b^ dimer) or the irrelevant I-A^b^ dimer (Mulv env145-158/I-A^b^ dimer) at a dose of 1 µg per mouse per day for 4 days. In addition, considering that soluble myelin peptide is a known means to achieve antigen-specific tolerization [18], the excess MOG35-55 peptide in a concentration used to load the dimer was also added as control. As shown in Fig. 2A, treatment with MOG35-55/I-A^b^ dimer reduced the clinical severity of EAE and arrested the progression of the disease throughout the observation period, while the mice received Mulv env145-158/I-A^b^ dimer or excess MOG35-55 peptide developed severe paralytic EAE as PBS control mice. The average cumulative disease index (CDI) of EAE in PBS control group, Mulv env145-158/I-A^b^ dimer-treated group or excess MOG35-55-treated group was higher than 30, which significantly decreased to less than 10 (9.25±5.6, n = 10) in MOG35-55/I-A^b^ dimer-treated group. There was also remarkable difference in peak disease score between mice treated with MOG35-55/I-A^b^ dimer and those in control groups (1.6±0.7 for MOG35-55/I-A^b^ dimer-treated group vs. 3.6±0.5 for PBS-treated group, 3.9±0.6 for Mulv env145-158/I-A^b^ dimer-treated group or 3.6±0.7 for excess MOG35-55-treated group, *P*<0.01). These data suggest that MOG35-55/I-A^b^ dimer treatment can delay onset and ameliorate EAE symptoms *in vivo*.

Pronounced cellular infiltration and demyelination are pathological hallmarks of EAE and MS. On day 25 post-immunization with MOG35-55 peptide, by H&E staining, marked multifocal and lymphohistiocytic inflammation that was both perivascular and diffuse was found in spinal cords from mice in PBS, excess MOG35-55 or Mulv env145-158/I-A^b^ dimer-treated group. In sharp contrast, most spinal cord samples from MOG35-55/I-A^b^ dimer-treated mice showed significantly fewer infiltrating cells (Fig. 2B upper panel). Luxol fast blue (LFB) staining for myelin loss analysis also revealed less damage to myelin sheets in mice treated with MOG35-55/I-A^b^ dimer than in those treated with PBS, excess MOG35-55 or Mulv env145-158/I-A^b^ dimer (Fig. 2B lower panel). Quantitative analysis of histological indices from 4 mice in each group showed that both inflammation and demyelination were ameliorated in mice treated with MOG35-55/I-A^b^ dimer compared to PBS control group (H&E: 1.3±0.5 vs. 3.0±0.8 [*P<*0.05, n = 4]; LFB: 1.5±0.6 vs. 4.0±0.8 [*P<*0.01, n = 4]) (Fig. 2C). No significant reduction of inflammation or demyelination was observed in Mulv env145-158/I-A^b^ dimer-treated mice. These results suggest that the striking therapeutic effects of MOG35-55/I-A^b^ dimer are antigen-specific and are reproducible at low dose.

### *In vivo* Administration of MOG35-55/I-A^b^ Dimer Decreases IFN-γ- and IL-17-Producing Cells in MOG35-55 Peptide-induced EAE Mice

**Figure 3:**
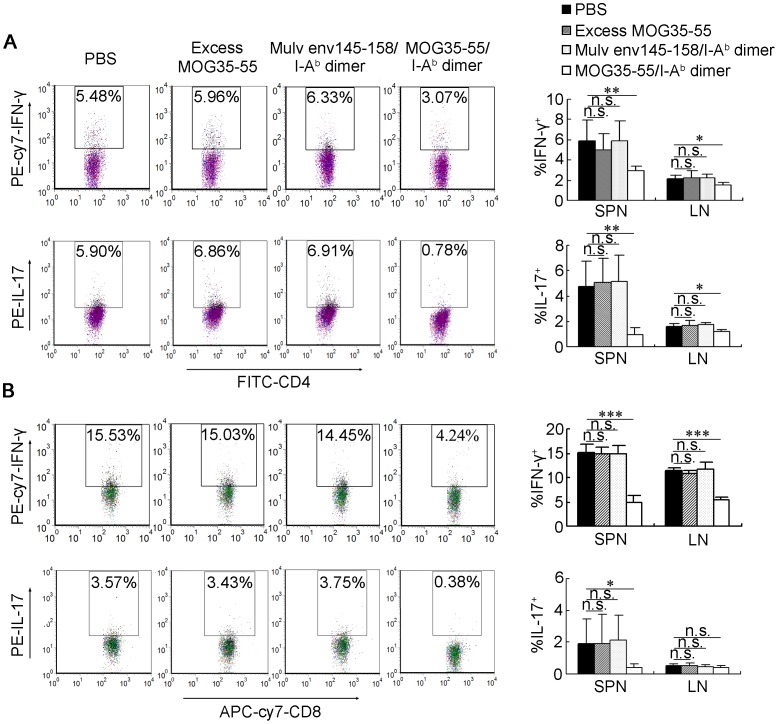
MOG35-55/I-A^b^ dimer treatment abrogates autoreactive Th1 and Th17 expansion *in vivo*. MOG35-55 immunized mice were treated with PBS, excess MOG35-55 peptide, Mulv env145-158/I-A^b^ dimer or MOG35-55/I-A^b^ dimer. On day 18 post-immunization, cells were isolated from spleen (SPN) and lymph nodes (LN) for IFN-γ^+^ Th1 and IL-17^+^ Th17 cells detection by intracellular staining. (A) Gated for CD3^+^CD4^+^ (B) Gated for CD3^+^CD8^+^. The left panel shows one representative sample from each group. The right panel represents the mean ± SD of percentage of Th1 or Th17 cells with differences (n≥4). *, *P<*0.05; **, *P<*0.01; ***, *P<*0.001 and n.s., nonsignificant.

Since Th1 and Th17 cells had been suggested to be the main effectors in disease pathogenesis in EAE**,** we further evaluated whether MOG35-55/I-A^b^ dimer treatment *in vivo* also abrogated the pathogenic Th1 and Th17 responses. Mice were sacrificed at the peak of actively induced EAE (day 18 after immunization) for detection of IFN-γ and IL-17 by flow cytometry. In EAE mice induced by MOG35-55 peptide immunization, the frequencies of IFN-γ-producing Th1 and IL-17-producing Th17 cells in the spleen were 5.86%±2.11% and 4.72%±2.01% (n = 6), respectively; MOG35-55/I-A^b^ dimer treatment significantly reduced the percentage of Th1 cells to 2.91%±0.44% (*P<*0.01, n = 6) (Fig. 3A, upper panel) and percentage of Th17 cells to 0.96%±0.53% (*P<*0.01, n = 6) (Fig. 3A, lower panel) in the spleen. Although the MOG35-55/I-A^b^ dimer was constructed to target CD4^+^ T cells specific for the MOG35-55 peptide, IFN-γ-producing CD8^+^ T cells were also markedly reduced in the spleens of MOG35-55/I-A^b^ dimer-treated mice (4.99%±1.42% for MOG35-55/I-A^b^ dimer-treated group vs. 15.20%±1.53% for PBS control [*P<*0.001, n = 6]) (Fig. 3B). The similar inhibitory effects of the MOG35-55/I-A^b^ dimer were also observed in the peripheral lymph nodes (LN) (Fig. 3). Importantly, the dimer-mediated abrogation of these pathogenic T cells was antigen-specific, as the control Mulv env145-158/I-A^b^ dimer-treated mice did not exhibited restraint on outburst of Th1 and Th17 cells, which was consistent with the serious clinical symptoms of EAE. In conclusion, our data suggest that not only the pathogenic Th1 and Th17 cells but also the IFN-γ- and IL-17-secreting CD8^+^ T cells in EAE mice can be significantly reduced by MOG35-55/I-A^b^ dimer treatment *in vivo*.

### MOG35-55/I-A^b^ Dimer Intervention Abrogates Pathogenic T cell Response by Downregulating TCR-CD3 Expression and Upregulating mTGF-β and IL-10 Production

**Figure 4:**
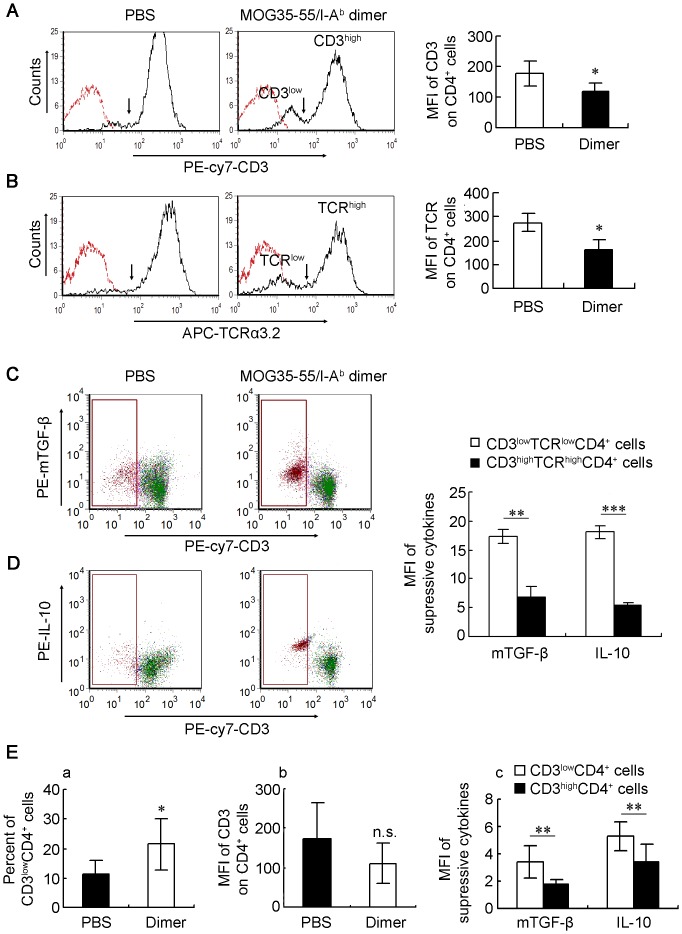
MOG35-55/I-A^b^ dimer modulates the expression of CD3 and TCR as well as the mTGF-β1 and IL-10 suppressive cytokines in MOG35-55-specific T cells. (A–D) The 2D2 splenocytes were stimulated with MOG35-55 (25 µg/mL) *in vitro* in the presence of MOG35-55/I-A^b^ dimer (1.2 nM) or PBS. After 5 days culture, cells were harvest and stained with anti-CD4-FITC mAb, anti-CD3-PE-Cy7 mAb, anti-TCR-Vα3.2-APC mAb, anti-mTGF-β1-PE and/or anti-IL-10-PE mAb. (A–B) The MFIs of (A) PE-cy7-CD3 and (B) APC-TCR Vα3.2 on CD4^+^ cells were analyzed by flow cytometry and expressed as the mean ± SD from 6 mice in each group. (C-D) MOG35-55/I-A^b^ dimer intervention induced a population of CD3^low^TCR^low^CD4^+^ cells with high level of (C) mTGF-β1 and (D) IL-10 expression. The right panel represents the mean ± SD of MFI of PE-mTGF-β1 and PE-IL-10 on CD3^high^TCR^high^CD4^+^/CD3^low^TCR^low^CD4^+^ cells in dimer intervention group (n = 3). (E) Splenocytes were isolated from mice treated with PBS or MOG35-55/I-A^b^ dimer on day 18 post-immunization and stained with anti-CD3-APC, anti-CD4-FITC, anti-TGF-β1-PE, and/or anti-IL-10-PE Abs. CD4^+^ cells were gated for analysis by flow cytometry. (a–b) The mean percentage of CD3^low^CD4^+^ cells and the MFI of APC-CD3 on CD4^+^ cells from 6 mice in each group are represented as the mean ± SD. (c) The MFIs of PE-mTGF-β1 and PE-IL-10 on CD3^low^CD4^+^/CD3^high^CD4^+^ cells in dimer treated-group are expressed as the mean ± SD from 6 mice respectively. *, *P<*0.05; **, *P<*0.01; ***, *P<*0.001 and n.s., nonsignificant.

Although Th2 cells and FoxP3^+^ Tregs have been reported to be critical for maintaining immune tolerance in autoimmune diseases, no significant increase of IL-4-producing or FoxP3 expression CD4^+^ cells was found in MOG35-55/I-A^b^ dimer-treated mice (Table S1). To test whether any other suppressive mechanisms were involved in the MOG35-55/I-A^b^ dimer-mediated inhibition of autoreactive T cell response, IL-10 and mTGF-β1 expressed by 2D2 CD4^+^ T cells were detected. After 5 days stimulation with MOG35-55 peptide in the presence/absence of MOG35-55/I-A^b^ dimer, the cells were harvested and stained with anti-Vα3.2 TCR-APC, anti-CD3-PE-cy7, anti-CD4-FITC, anti-TGF-β1-PE, and/or anti-IL-10-PE Abs. The mean florescence intensity (MFI) was measured to indicate the molecules expression levels. We found that MOG35-55/I-A^b^ dimer intervention induced significant downregulation of TCR-CD3 expression on CD4^+^ MOG35-55 peptide-reactive 2D2 T cells. Specifically, the CD3 and Vα3.2 TCR expression on CD4^+^2D2 T cells were decreased in the MOG35-55/I-A^b^ dimer intervention group compared with the PBS control group in terms of MFI (CD3 MFI: 162.94±41.45 vs. 274.72±36.83 [*P<*0.05, n = 6]; TCR MFI: 162.94±41.45 vs. 274.72±36.83 [*P<*0.05, n = 6]) (Fig. 4A–B). Based on the CD3 and TCR expression levels (CD3 MFI cutoff = 50; TCR MFI cutoff = 60), the MOG35-55 peptide-activated T cells can be divided into 2 subsets: those with lower expression of CD3 and TCR (CD3^low^TCR^low^CD4^+^ cells) and those with higher expression of CD3 and TCR (CD3^high^TCR^high^CD4^+^ cells). The percentage of CD3^low^TCR^low^CD4^+^ cells increased to 22.80%±5.47% (n = 6) in the MOG35-55/I-A^b^ dimer intervention group, as compared to only about 8% in the PBS control group. Furthermore, this expanded subset of CD3^low^TCR^low^CD4^+^ cells had higher levels of mTGF-β1 and IL-10 than the other CD4^+^ subset. The MFI of mTGF-β1 on CD3^low^TCR^low^CD4^+^ cells was at least twice as high as that of the CD3^high^TCR^high^CD4^+^ cells after dimer intervention (18.75±5.69 vs. 6.85±2.89) (Fig. 4C); similar results were observed in the IL-10 expression (MFI: 18.03±1.39 vs. 9.25±1.74) (Fig. 4D).

To confirm these findings *in vivo*, the MOG35-55-induced EAE mice treated with PBS or MOG35-55/I-A^b^ dimer were sacrificed at the peak of actively induced EAE (day 18 after immunization), and splenocytes were isolated and stained with anti-CD3-APC, anti-CD4-FITC, anti-TGF-β1-PE, and/or anti-IL-10-PE Abs for detection by flow cytometry. Consistent with the *in vitro* results, treatment with MOG35-55/I-A^b^ dimer *in vivo* induced an increased percentage of CD3^low^CD4^+^ T cells compared with PBS control mice (21.50%±8.73% vs. 11.52%±4.60%, [P<0.05,n = 6]) (Fig. 4Ea), although the downregulation of CD3 MFI on CD4^+^ cells was not significant (110.98±52.49 vs. 174.27±91.83, [P = 0.08,n = 6]) (Fig. 4Eb). Furthermore, the MFIs of mTGF-β1 and IL-10 on this CD3^low^CD4^+^ T cell population were also significantly higher than those on CD3^high^CD4^+^ cells (mTGF-β1 MFI: 3.45±1.16 vs. 1.72±0.38, [P<0.01,n = 6]; IL-10 MFI: 5.31±1.08 vs. 3.36±1.39, [P<0.01,n = 6]) (Fig. 4Ec), Taken together, these results suggest that the outgrowth of CD3^low^TCR^low^CD4^+^ cells with high levels of mTGF-β and IL-10 suppressive cytokine production may be one mechanism the soluble MOG35-55/I-A^b^ dimer uses to suppress autoreactive T cells.

## Discussion

Using the MOG35-55 peptide-induced EAE model in C57BL/6 mice, our study showed that the MOG35-55/I-A^b^ dimer can inhibit autoreactive T-cell expansion, reduce inflammation in CNS, and ameliorate clinical signs of EAE. We proposed that this dimer treatment was successful because binding of TCR ligand to T cells without co-stimulatory signals could lead to anergy of the antigen-specific T cells; in effect, we exploited this strategy to restore peripheral tolerance. In previous studies, monomeric class I and II MHC molecules were generated to target and bind antigen-specific T cells with low binding affinity, but without causing TCR multimerization [8], [19], [20]. While divalent peptide/MHC molecules initially triggers T-cell activation through causing TCR multimerization, the effect of the peptide/MHC dimer is to dampen pathogenic T cells because this response is followed by a state of unresponsiveness that is refractory to restimulation [19]. In the EAE treatment field specifically, monomeric RTLs lacking the CD4-binding β2 domain have been shown to cause a unique pattern of downstream activation to effectively reduce the encephalitogenic activity of the T cells [21], [22], [23], [24]. The MOG35-55/I-A^b^ dimer constructed in this paper was made of bivalent TCR ligands dimerized by the disulfide bonds around the hinge region of murine IgG Fc fragments. A relatively low dose of MOG35-55/I-A^b^ dimer (1 µg of the dimer per mouse daily for 4 days) achieved improved symptom-score in the MOG35-55 peptide-induced EAE mouse, indicating that the soluble peptide/MHC dimer may offer another promising therapeutic choice for EAE and MS, as well as other T-cell mediated autoimmune diseases.

Due to the fact that the MOG35-55/I-A^b^ dimer can downregulate CD3-TCR expression on autoreactive T cells, it shares some similarities with anti-CD3 or anti-TCR antibody treatment; the latter has previously been assessed for mouse EAE and human MS treatment [25], [26]. The antibody-ligation-mediated TCR-CD3 downregulation is responsible for T-cell immune dysfunction or T-cell tolerance. The numbers of TCR on one T cell range from 30,000 to 100,000 [27]. It has been well-demonstrated that very few surface TCR molecules (1000 or less) were needed for T cells to respond to immunization and a 95% reduction in TCR expression does not adversely affect response to foreign Ags [27], [28]. However, self-reactive T cells have been found to be affected by a small reduction in TCR expression [29]. This sensitivity to incremental TCR reduction is due to self-Ag likely acting as a weak ligand, and self-reactive T cells do not have spare receptors on their surface. Indeed, they require their full contingent of TCRs expressed on the surface to respond to their weak cognate ligands. This might explain how the 40% TCR and 33% CD3 reduction on the surface of the 2D2 CD4^+^ T cells by the MOG35-55/I-A^b^ dimer intervention may have led to more than 90% inhibition of T cell proliferation (Figs. 1, 4). The TCR-CD3 downregulation would also account for the diminishing encephalitogenic Th1 and Th17 cell percentage both *in vitro* and *in vivo* (Figs. 1, 3), as antigen recognition by TCR followed by activation of the TCR-mediated signaling pathways are the most critical steps to initiate Th cells differentiation [30], [31]. The strategy using soluble peptide/MHC complexes as antigen-specific modulators–due to their specificity for cognate TCR–is more desirable than anti-CD3 or anti-TCR treatment strategy, as global depletion and potent activation of T cells by these antibodies demonstrated less impressive results in MS patients and EAE animal models during initial trials [25], [26]. In this study, MOG35-55/I-A^b^ dimer treatment induced a significant amelioration of EAE and diminished the frequencies of encephalitogenic Th1 and Th17 cells, while I-A^b^ dimer loaded with an irrelevant peptide did not show the therapeutic effect, suggesting the soluble dimer acted in an antigen-specific manner (Figs. 2, 3).

While we observed that IFN-γ- and/or IL-17-producing CD8^+^ T cells significantly expanded in the MOG35-55 peptide-induced EAE control mice, the percentage of these cells was significantly reduced in MOG35-55/I-A^b^ dimer-treated EAE mice (Fig. 3B) even though the MOG35-55/I-A^b^ dimer was designed to directly target the CD4^+^ autoreactive T cells. When we tested the ability of a soluble MHC class I dimer molecule (MOG35-55/H-2D^b^ dimer) to specifically target and inhibit MOG35-55-specific CD8^+^ T cells, it failed to inhibit CD8^+^ encephalitogenic T cells *in vitro* (Fig. S2). These results may suggest the relative importance of CD4^+^ and CD8^+^ T cells and their interplay in EAE and MS in the following ways. First, CD4^+^ T cells play an almost exclusive role throughout the disease progression of EAE and MS. Indeed, even though CD8^+^ T cells are recognized to be responsible for disease exacerbation, especially in the late stages of disease, strategies that target CD8^+^ T cells are insufficient to reverse EAE. Second, EAE therapies that target CD4^+^ T cells also influence the function of CD8^+^ T cells, probably through regulating the CD4^+^ Th cell network.

Our work suggests that the therapeutic effect of the MOG35-55/I-A^b^ dimer on EAE mice resulted from the induction of T cells with regulatory properties. The MOG35-55 peptide-activated 2D2 T cells with intervention of this dimer *in vitro* were tested for suppressive markers of FoxP3, IL-10 and mTGF-β1. Interestingly, we found that the same MOG peptide-specific T cells that downregulated CD3-TCR expression exhibited upregulation of mTGF-β1 and IL-10. Consistent with our *in vitro* results, we also observed an increased population of CD3^low^CD4^+^ T cells with high levels of mTGF-β1 and IL-10 in the peripheral lymphoid organs in MOG35-55/I-A^b^ dimer-treated WT EAE mice, although the decrease in CD3 MFI was not as apparent as in the *in vitro* experiments (Fig. 4E). Two types of Tregs have been proposed to play vital role in immune homeostasis and protection against autoimmunity. One type is the natural Treg (nTreg) cell with a CD4^+^CD25^+^FoxP3^+^ phenotype, which is selected by high-avidity interactions in the thymus. The other type is the adaptive Treg, which develops outside of the thymus under subimmunogenic antigen presentation, including IL-10-secrecting type 1 T regulatory (Tr1) cells, TGF-β-secreting Th3-type regulatory T cells and etc. [32], [33]. Although the origin and the transformation relationship among different phenotypes of adaptive Tregs are still not clear, it is well accepted that the two inhibitory cytokines: TGF-β and IL-10 play key role in mediating the immunosuppressive effect and are considered to be typical markers of adaptive Tregs [34], [35]. Although nTregs with enhanced FoxP3 expression have been considered as important contributors to remain suppression of T effector/autoreative cells [36], we did not observe a significant increase in CD4^+^CD25^+^Foxp3^+^ Tregs *in vitro* and *in vivo*. Instead, T cells with an adaptive Treg phenotype (TGF-β^+^IL10^+^CD4^+^FoxP3^-^) expanded dramatically after dimer intervention. The outgrowth of CD3^low^TCR^low^CD4^+^ T-cell population with high mTGF-β1 and IL-10 expression suggests that both TGF-β- and IL-10-dependent regulatory mechanisms are involved in the MOG35-55/I-A^b^ dimer-induced suppression.

This study demonstrated that the MOG35-55/I-A^b^ dimer can reduce encephalitogenic T cell numbers and ameliorate EAE symptoms induced by the MOG35-55 peptide in C57BL/6 mice. The efficacy of dimer treatment was both antigen-specific and efficient. Furthermore, dimer intervention promoted the outgrowth of antigen-specific T cells with a regulatory phenotype exhibiting downregulated TCR-CD3 expression and upregulated mTGF-β1 and IL-10 expression. Although our findings suggest that soluble MHC class II dimer can be used clinically to treat autoimmune disease, human MS is much more complicated than the MOG-induced EAE mouse model, where there is only a single self-peptide involved and the MHC allele is known. An extensive understanding of the MS-related self-antigens and their cognate T-cell epitopes restricted by the diverse MHC alleles present in the random population is required before this soluble MHC dimer strategy can be used clinically to treat human MS.

## Materials and Methods

### Ethics Statement

Animal experiments in this study were approved by the Ethical Committee on Animal Experimentation of Tongji Medical College, Huazhong University of Science and Technology, China (Approval ID: 00018570).

### Mice

Female C57BL/6 mice at 6–8 weeks old were purchased from the Chinese Shanghai Laboratory Animal Center (Shanghai, China). The 2D2 transgenic mice, expressing Vα3.2/Vβ11 TCR specific to MOG35-55 peptide, on the C57BL/6 background were purchased from Jackson Laboratory (Bar Harbor, ME) [6]. The mice were bred and maintained under specific pathogen-free conditions and treated in accordance with Tongji Medical College (China) animal care guidelines.

### Peptide

I-A^b^-restricted self-origin peptide MOG35-55 (MEVGWYRSPFSRVVHLYRNGK), I-A^b^-restricted irrelevant peptide Mulv env145-158 (HNEGFYVTPGPGRP) and I-E^b^-restricted irrelevant peptide BSA141-158 (GKYLYEIARRHPYF) [37] were synthesized by a peptide synthesizer and purified to >98% homogeneity by reverse-phase HPLC. The peptides were dissolved in DMSO and diluted to 1mg/mL with RPMI-1640 medium (Gibco).

### Preparation of the I-A^b^/IgG Chimeric Protein and Peptide Pulsing

Construction and purification of I-A^b^ dimer were performed using a previously published protocol [38]. The cDNA coding domain for the extracellular domains of I-A^b^α (α1-α2) and I-A^b^β (β1-β2) as well as the Fos and Jun dimerization motifs were amplified with reverse-transcription polymerase chain reaction (RT-PCR) from C57BL/6 splenocytes. The Fc segment, spanning the hinge region and the CH2 and CH3 domains of murine IgG2b were amplified from C57BL/6 splenocytes. I-A^b^α-Fos-Fc and I-A^b^β-Jun segments were cloned into two multiple clone sites of the baculovirus expression vector pFastBac^TM^Dual (Invitrogen, Cat#10712-024). A recombinant baculovirus was generated using the Bac-to-Bac Baculovirus Expression system (Invitrogen, Cat#10359-016). The dimer was produced in sf9 cells (Invitrogen, Cat#11496-015) cultured in SF-900 II SFM media (Gibco, Carlsbad, CA, U.S., Cat#10902-104), and the recombinant baculovirus was infected into sf9 cells in the exponential stage of growth at 1.5×10^6^ cells/mL. Infected cells were incubated at 27°C for approximately 5 days until 70–80% of the cells were dead. The supernatants were harvested and purified by affinity chromatography using a protein A column (Invitrogen, Cat#15918-014). The purified dimer was then dialyzed against pH 7.4 phosphate-buffered saline (PBS) and stored at −80°C before use. The purified dimer was stored at a 12 nM concentration (≈2 µg/mL) and was pulsed with the self-peptide MOG35-55 to form the MOG35-55/I-A^b^. To pulse the dimer with peptide, the fusion protein was incubated with excess peptide at a mol ratio of 1∶640 at 37°C for 24–48 h. The dimer pulsed with MOG35-55, Mulv env145-158 or BSA141-158 is named as MOG35-55/I-A^b^ dimer, Mulv env145-158/I-A^b^ dimer or BSA141-158/I-A^b^ dimer (mismatched), respectively. Another set of I-A^b^ dimers pulsed with corresponding biotinylated peptides was set up to verify the effectively loading of peptide on the MHC class II dimer. The three peptides had been biotinylated by the EZ-Link®Sulfo-NHS-SS-Biotin kit (Thermo Scientific, Cat#21331) according to the instruction book, and loaded onto I-A^b^ dimer as described above to form biotinylated-peptide/I-A^b^ dimers.

### Detection of the Dimer by ELISA and Western Blotting

The soluble I-A^b^ dimer was detected by sandwich ELISA with mouse MHC-class-II-(I-A/I-E)-specific mAb: 2G9 (BD, Cat#553621) or M5/114.15.2 (BD, Cat#556999) as capture antibody, and HRP-labeled mouse IgG2b-specific mAb LO-MG2b-2 (SouthernBiotech, Cat#1185-05) as detected antibody; The biotinylated-peptide/I-A^b^ dimers were detected with an IgG2b-specific mAb LO-MG2b-2 and HRP-labeled streptavidin (Boster, Cat#BA1088). The plate was developed with ortho-phenylenediamine (Sigma, Cat#P-8412) and absorbance was read at 492 nm. For quantification, soluble I-A^b^ dimer was also detected with goat anti-mouse IgG as a primary antibody and HRP-labeled rat anti-mouse IgG as the secondary antibody. Reference curves were performed using serial dilutions of calibrated mouse IgG (Sigma, Cat#I-5381).

The protein A column-purified I-A^b^ dimer was fractionated in 12% SDS-PAGE, and electrophoretically transferred to a nitrocellulose membrane, then detected by rabbit anti-c-Fos (Boster, Cat#BA0207) or rabbit anti-c-Jun antibody (Boster, Cat#BA0208). HRP-conjugated anti-rabbit IgG antibody (eBioscience, Cat#18-8816) was used as the secondary antibody.

### *In vitro* Activation of MOG35-55-specific Autoreactive T cells

Single cell suspensions of mononuclear cells were isolated by density gradient centrifugation (Ficoll-Hypaque, density 1.077 g/mL) from 2D2 transgenic mice or MOG35-55-immunized wild-type C57BL/6 mice splenocytes upon onset of actively induced EAE (day 9 after immunization). The mononuclear cells were then cultured in 10% FCS RPMI-1640 medium. 3×10^6^ splenocytes were then cultured in 24-well flat-bottom plates with 25 µg/mL MOG35-55 and 20 U/mL IL-2. MOG35-55/I-A^b^ dimer, excess MOG35-55 peptide or PBS was added into this culture, and the cells were harvested after 3 days for T cell proliferation assay and 5 days for phenotype analysis.

### T cell Proliferation Assay

T cell proliferation was measured by fluorescent dye dilution. The lymphocytes were labeled with 2 µM carboxyfluorescein diacetate succinimidyl ester (CFSE) (Sigma, Cat#C5041) before culturing. After 3 days of culture, cells were harvested and incubated on ice with anti-CD3-APC (eBioscience, Cat#17-0031-82) and anti-CD4-PE (eBioscience, Cat#12-0041-82) antibodies for 1 h. They were then washed and fixed with PBS containing 2% formaldehyde. Three-color analysis was performed with CFSE, anti-CD3-APC, and anti-CD4-PE using a LSRII flow cytometer (BD Biosciences). Flow cytometric data files were analyzed with the Proliferation Wizard module in the ModFit LT Macintosh software (BD Biosciences).

### Flow Cytometry and Intracellular Cytokine Staining

T cell phenotype was analyzed by a FACS LSRII system (BD Biosciences). The following fluorescence-conjugated mAbs used in this study were purchased from eBioscience: anti-Vα3.2 TCR-APC (Cat#17-5799-82), anti-CD3-PE-cy7 (Cat#25-0031-82), anti-CD3-APC (Cat#17-0031-82), anti-CD4-FITC (Cat#11-0041-82), anti-CD25-PE-cy7 (Cat#25-0251-82), anti-CTLA-4-PE (Cat#12-1522-82), anti-IFN-γ-PE-cy7 (Cat#25-7311-82), anti-IL-17-PE (Cat#12-7177-81), anti-IL-4-PE-cy7 (Cat#25-7042-42), and anti-IL-10-PE (Cat#12-7101-82). Anti-CD8-APC-cy7 (Cat#100714) and anti-TGF-β1-PE (Cat#141404) were obtained from Biolegend. For intracellular staining, cells were stimulated for 4 h with 50 ng/mL phorbol 12-myristate 13-acetate (PMA, Sigma) and 500 ng/mL ionomycin (Sigma) in the presence of Brefeldin A (GolgiStop, eBioscience). After staining with antibodies against surface markers, cells were fixed with IC Fixation Buffer (eBioscience), permeabilized using 1×permeabilization buffer (eBioscience), and lastly incubated with antibodies against intracellular cytokines. For intracellular FoxP3 staining, the mouse/rat FoxP3 staining kit (eBioscience, Cat#72-5775-40) was used according to the manufacturer’s instructions.

### Induction of Active EAE and Treatment with MOG35-55/I-A^b^ Dimer

Female C57BL/6 mice were subcutaneously (s.c.) immunized with 200 µL of an emulsion containing 200 µg of MOG35-55 and an equal volume of complete Freund’s adjuvant (CFA), which contained 4 mg/mL of heat-killed *Mycobacterium tuberculosis* strain H37Ra (Difco, MI), over the 4 flanks. Pertussis toxin (250 ng) (Sigma) in 0.2 mL PBS was given intraperitoneally (i.p.) on the day of immunization as well as 48 h later. Mice were monitored daily for clinical signs of disease and were scored as follows: 0, no signs; 1, limp tail and ataxia; 3, paralysis of one hind limb; 4, complete hind limb paralysis; and 5, moribundity or death.

Mice were divided into four groups (n = 8–10 for each group) and treated i.p. with 500 µl PBS, 500 µl excess MOG35-55 peptide in a concentration used to load the dimer, 500 µl of 12 nM irrelevant dimer (Mulv env145-158/I-A^b^ dimer), or 500 µL of 12 nM experimental dimer (MOG35-55/I-A^b^ dimer) at a dose of 1 µg per mouse per day for 4 days. Animals were monitored for changes in disease score until they were euthanized for histopathology on day 25 post-immunization. Alternatively, mice were sacrificed at the peak of actively induced EAE (day 18 after immunization), and mononuclear cells from spleens and draining lymph nodes were harvested for flow cytometry analysis of IFN-γ, IL-17, mTGF-β1, IL-10, IL-4, and/or FoxP3.

### Histopathology

Four randomly chosen mice from the PBS, excess MOG35-55, Mulv env145-158/I-A^b^ dimer, and MOG35-55/I-A^b^ dimer treatment groups were perfused with 0.9% saline followed by cold 4% paraformaldehyde. Spinal cords were removed and post-fixed in 4% paraformaldehyde for 48 h. The spinal cords were dissected after fixation and embedded in paraffin before sectioning. The sections were stained with Luxol fast blue (LFB) or periodic acid-Schiff-hematoxylin to assess demyelination and inflammatory lesions. Manual tracing was used to define the degree of inflammation and demyelination across each entire spinal cord section. Pathological changes in each spinal cord were scored as follows: 0, no change; 1, focal area involvement; 2, <5% of total myelin area involved; 3, 5–10% of total myelin area involved; 4, 10–20% of total myelin area involved; 5, >20% of total myelin area involved [39]. Demyelination and inflammatory scores were determined independently by two investigators and reviewed for consensus in cases of scoring differences.

### Statistical Analysis

Statistical differences among disease and pathological scores of mice treated with PBS, excess MOG35-55, Mulv env145-158/I-A^b^ dimer and MOG35-55/I-A^b^ dimer were evaluated using the Mann-Whitney U test. Other data were analyzed for statistical significance by ANOVA test using SPSS15.0 for Windows software. *P<*0.05 was considered statistically significant. The significant differences were marked as *, 0.01<*P<*0.05; **, 0.001<*P<*0.01; and ***, *P<*0.001, respectively.

## Supporting Information

Figure S1 **Construction, expression and peptide pulsing of the I-A^b^ dimer.** (A) Schematic representation of genetic construction of pFastBac^TM^Dual+[I-A^b^/Fc] plasmid. The I-A^b^α-Fos-Fc gene was constructed by fusing the extracellular domain of I-A^b^α chain with Fos gene and the Fc portion of IgG2b at the C terminal end. The I-A^b^β-Jun fusion gene was constructed by covalently attaching the Jun gene to the C terminal of the extracellular domain of I-A^b^. The two fusion genes (I-A^b^α-Fos-Fc and I-A^b^β-Jun) were cloned into double expression plasmid pFastBac^TM^Dual at downstream of promoters PH and P10, respectively, generating the recombinant plasmid. (B) Model of the proposed structure of the I-A^b^ dimer. (C) Detection of the I-A^b^ dimer by sandwich ELISA with an I-A/I-E conformation-specific mAb (2G9 or M5.114.15.2) and rat anti-mouse IgG2b mAb. Each sample was tested in nonuple and the results are presented as mean ± SD of OD492. (D) Reduced SDS-PAGE (lane 1) and western blotting with polyclonal antibodies specific for Fos (lane 2) and Jun (lane 3) show the purified I-A^b^ dimer consists of two bands with molecular mass corresponding to I-A^b^α-Fos-Fc (53 kD) and I-A^b^β-Jun (31 kD). (E) Detection of the biotinylated-peptide/I-A^b^ dimers with IgG2b-specific mAb and HRP-labeled streptavidin: (a) PBS; (b) MOG35-55/I-A^b^ dimer (not biotinylated); (c) biotinylated-BSA141-158/I-A^b^ dimer (mismatched); (d) biotinylated-MOG35-55/I-A^b^ dimer; (e) biotinylated-Mulv env145-158/I-A^b^ dimer. Each sample was tested in nonuple and the results are presented as mean ± SD of OD492.
(TIF)

Figure S2 **Soluble MHC I MOG35-55/H-2D^b^ dimer does not inhibit CD8^+^ or CD4^+^ MOG35-55-specific T cells.** (A) Splenocytes from MOG35-55-immunized wild-type C57BL/6 mice were stimulated with MOG35-55 (25 µg/mL) *in vitro*. Intracellular staining of cells for IFN-γ and (B) IL-17 was performed after 5 days culture in four different groups: (a) PBS control; (b) MOG35-55/H-2D^b^ dimer (1.2 nM); (c) MOG35-55/I-A^b^ dimer (1.2 nM); (d) MOG35-55/H-2D^b^ dimer (1.2 nM) + MOG35-55/I-A^b^ dimer (1.2 nM). The mean percentage of positive cells is represented as the mean ± SD. The differences were shown by comparing the three dimer intervention groups with PBS control (n = 3). *, *P<*0.05; **, *P<*0.01.
(TIF)

Table S1 **Detection of common suppressive markers on CD4^+^ T cells after MOG35-55/I-A^b^ dimer administration** ***in vitro*** **and** ***in vivo*** **^a^.** ^a^ For *in vivo* research, wild-type C57BL/6 mice were immunized to induce active EAE and were treated with PBS or MOG35-55/I-A^b^ dimer at a dose of 1 µg per mouse per day for 4 days. Mice were sacrificed on day 18 after immunization, and splenocytes were collected; *in vitro*, the splenocytes of MOG35-55 immunized wild-type C57BL/6 mice were stimulated with MOG35-55 peptide (25 µg/mL). MOG35-55/I-A^b^ dimer (1.2 nM) or PBS was added and co-cultured with the cells for 5 d, and then the cells were harvested. IL-4-, IL-10-, FoxP3-, mTGF-β-, and CTLA4-positive cells were analyzed by flow cytometry. Data are expressed as means ± SD from at least 3 mice. *, *P<*0.05 vs. PBS control. ^b^ Dimer: MOG35-55/I-A^b^ dimer. ^C^ ND: not done.
(DOC)
