# Supplementary figures and images for: Soluble MOG35-55/I-Ab Dimers Ameliorate Experimental Autoimmune Encephalomyelitis by Reducing Encephalitogenic T Cells
Source: PLoS One. 2012 Oct 15;7(10):e47435. doi: 10.1371/journal.pone.0047435 (PMC3471819; doi:10.1371/journal.pone.0047435)

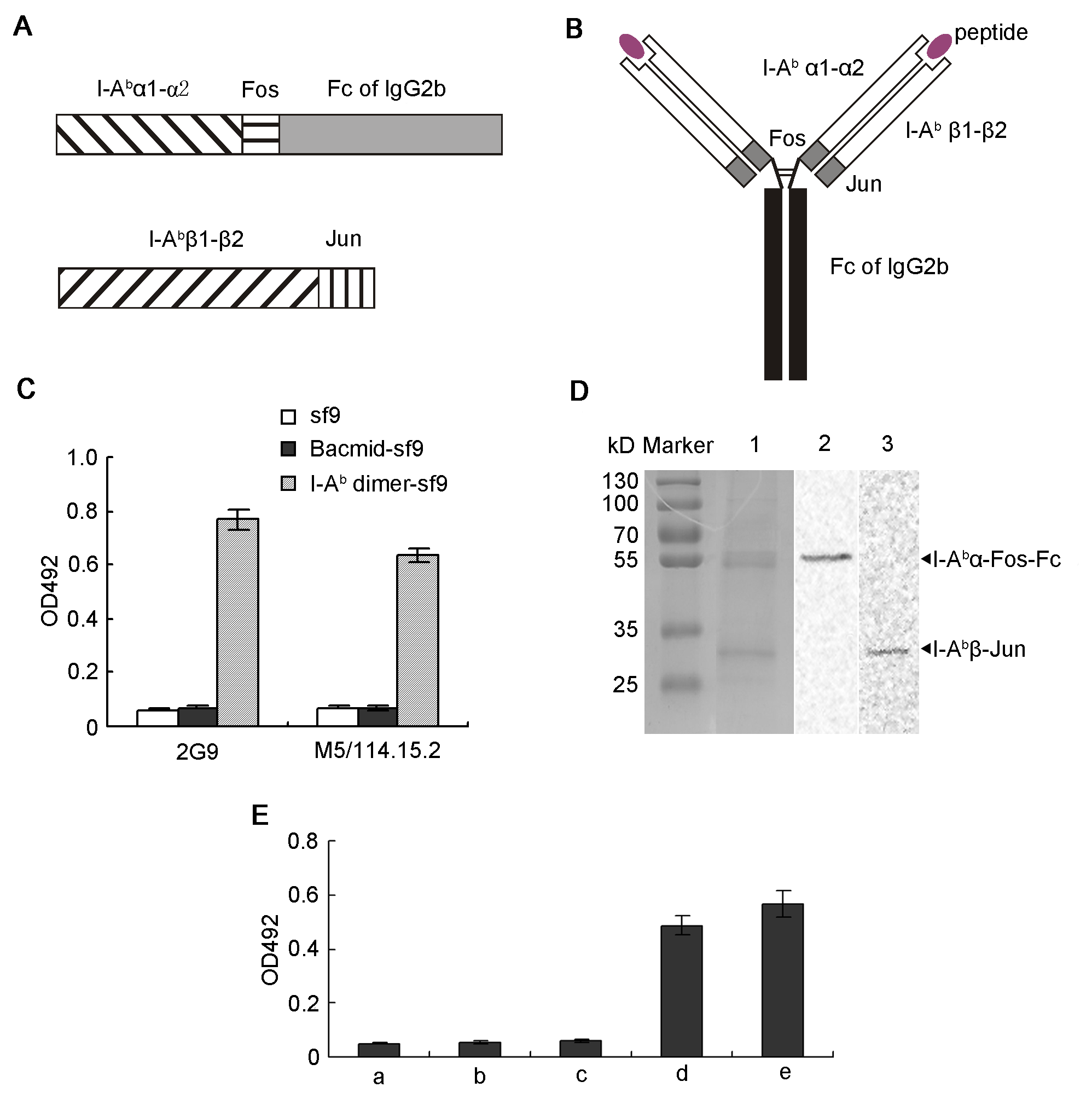

Supplement: Figure S1 — Construction, expression and peptide pulsing of the I-Ab dimer. (A) Schematic representation of genetic construction of pFastBacTMDual+[I-Ab/Fc] plasmid. The I-Abα-Fos-Fc gene was constructed by fusing the extracellular domain of I-Abα chain with Fos gene and the Fc portion of IgG2b at the C terminal end. The I-Abβ-Jun fusion gene was constructed by covalently attaching the Jun gene to the C terminal of the extracellular domain of I-Ab. The two fusion genes (I-Abα-Fos-Fc and I-Abβ-Jun) were cloned into double expression plasmid pFastBacTMDual at downstream of promoters PH and P10, respectively, generating the recombinant plasmid. (B) Model of the proposed structure of the I-Ab dimer. (C) Detection of the I-Ab dimer by sandwich ELISA with an I-A/I-E conformation-specific mAb (2G9 or M5.114.15.2) and rat anti-mouse IgG2b mAb. Each sample was tested in nonuple and the results are presented as mean ± SD of OD492. (D) Reduced SDS-PAGE (lane 1) and western blotting with polyclonal antibodies specific for Fos (lane 2) and Jun (lane 3) show the purified I-Ab dimer consists of two bands with molecular mass corresponding to I-Abα-Fos-Fc (53 kD) and I-Abβ-Jun (31 kD). (E) Detection of the biotinylated-peptide/I-Ab dimers with IgG2b-specific mAb and HRP-labeled streptavidin: (a) PBS; (b) MOG35-55/I-Ab dimer (not biotinylated); (c) biotinylated-BSA141-158/I-Ab dimer (mismatched); (d) biotinylated-MOG35-55/I-Ab dimer; (e) biotinylated-Mulv env145-158/I-Ab dimer. Each sample was tested in nonuple and the results are presented as mean ± SD of OD492. (TIF) [file pone.0047435.s001.tif]

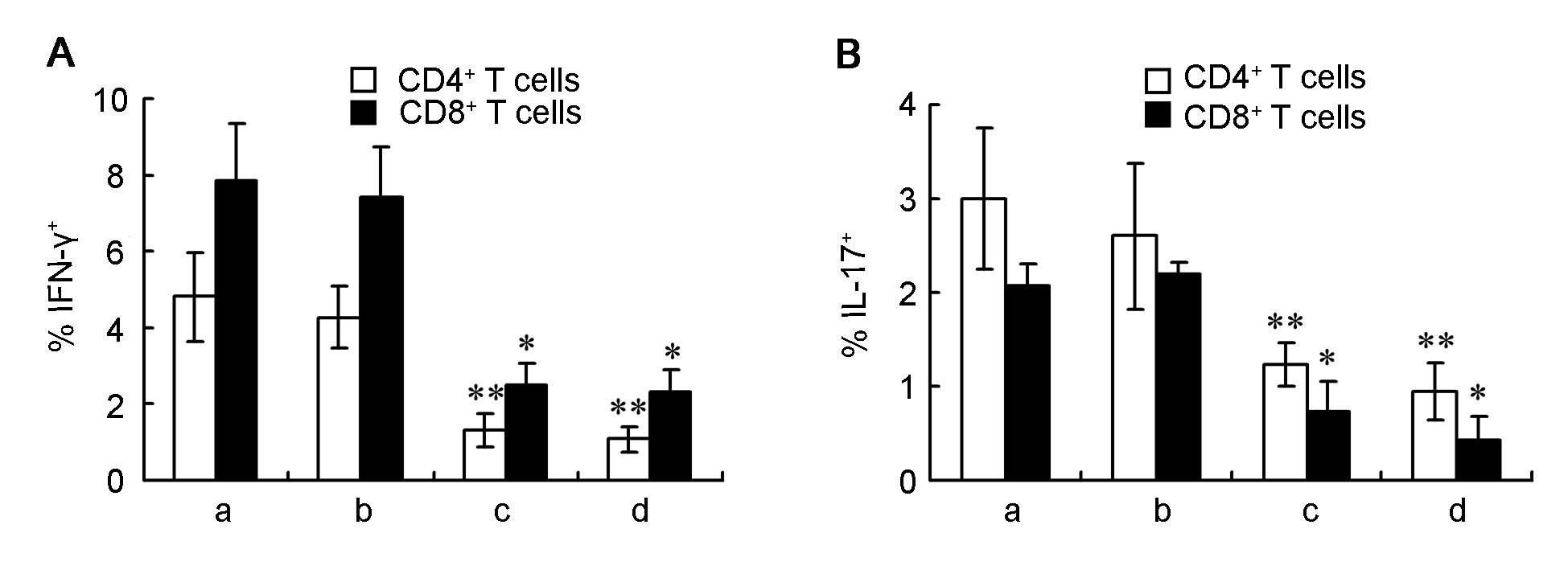

Supplement: Figure S2 — Soluble MHC I MOG35-55/H-2Db dimer does not inhibit CD8+ or CD4+ MOG35-55-specific T cells. (A) Splenocytes from MOG35-55-immunized wild-type C57BL/6 mice were stimulated with MOG35-55 (25 µg/mL) in vitro. Intracellular staining of cells for IFN-γ and (B) IL-17 was performed after 5 days culture in four different groups: (a) PBS control; (b) MOG35-55/H-2Db dimer (1.2 nM); (c) MOG35-55/I-Ab dimer (1.2 nM); (d) MOG35-55/H-2Db dimer (1.2 nM) + MOG35-55/I-Ab dimer (1.2 nM). The mean percentage of positive cells is represented as the mean ± SD. The differences were shown by comparing the three dimer intervention groups with PBS control (n = 3). *, P<0.05; **, P<0.01. (TIF) [file pone.0047435.s002.tif]
