# Supplementary material for: Soluble MOG35-55/I-Ab Dimers Ameliorate Experimental Autoimmune Encephalomyelitis by Reducing Encephalitogenic T Cells
Source: PLoS One. 2012 Oct 15;7(10):e47435. doi: 10.1371/journal.pone.0047435 (PMC3471819; doi:10.1371/journal.pone.0047435)
Supplement: Table S1 — Detection of common suppressive markers on CD4+ T cells after MOG35-55/I-Ab dimer administration in vitro and in vivo a. a For in vivo research, wild-type C57BL/6 mice were immunized to induce active EAE and were treated with PBS or MOG35-55/I-Ab dimer at a dose of 1 µg per mouse per day for 4 days. Mice were sacrificed on day 18 after immunization, and splenocytes were collected; in vitro, the splenocytes of MOG35-55 immunized wild-type C57BL/6 mice were stimulated with MOG35-55 peptide (25 µg/mL). MOG35-55/I-Ab dimer (1.2 nM) or PBS was added and co-cultured with the cells for 5 d, and then the cells were harvested. IL-4-, IL-10-, FoxP3-, mTGF-β-, and CTLA4-positive cells were analyzed by flow cytometry. Data are expressed as means ± SD from at least 3 mice. *, P<0.05 vs. PBS control. b Dimer: MOG35-55/I-Ab dimer. C ND: not done. (DOC) [file pone.0047435.s003.doc]

**Table S1. Detection of common suppressive markers on CD4+ T cells after MOG35-55/I-Ab dimer administration *in vitro* and *in vivo*a**

| Cytokine(%) | *In vivo* | |  | *In vitro* | |
| --- | --- | --- | --- | --- | --- |
|  | PBS | Dimer^b^ |  | PBS | Dimer^b^ |
| IL-10^+^/CD4^+^  IL-4^+^/CD4^+^  FoxP3^+^CD25^+^CD4^+^  mTGF-β^+^/CD4^+^  CTLA4^+^/CD4^+^ | 0.53±0.1  0.30±0.2  1.07±0.6  2.35±0.3  ND^c^ | 0.71±0.2  0.38±0.1  1.18±0.9  3.14±1.1  ND^c^ |  | 1.11±0.3  0.52±0.3  1.98±0.8  3.53±0.6  13.35±1.7 | 2.85±0.1*  1.09±0.9  2.38±0.4  7.74±3.9*  10.95±2.0 |

^a^ For *in vivo* research, wild-type C57BL/6 mice were immunized to induce active EAE and were treated with PBS or MOG35-55/I-A^b^ dimer at a dose of 1 μg per mouse per day for 4 days. Mice were sacrificed on day 18 after immunization, and splenocytes were collected; *in vitro*, the splenocytes of MOG35-55 immunized wild-type C57BL/6 mice were stimulated with MOG35-55 peptide (25 μg/mL). MOG35-55/I-A^b^ dimer (1.2 nM) or PBS was added and co-cultured with the cells for 5 d, and then the cells were harvested. IL-4-, IL-10-, FoxP3-, mTGF-β-, and CTLA4-positive cells were analyzed by flow cytometry. Data are expressed as means ± SD from at least 3 mice. *, *P<*0.05 vs. PBS control*.*

^b^ Dimer: MOG35-55/I-A^b^ dimer.

^C^ ND: not done.
